# Supplementary material for: From Food Waste to Sustainable Agriculture: Nutritive Value of Potato By-Product in Total Mixed Ration for Angus Bulls
Source: Foods. 2024 Aug 30;13(17):2771. doi: 10.3390/foods13172771 (PMC11394973; doi:10.3390/foods13172771)
Supplement: Supplementary file 1 [file foods-13-02771-s001.zip › foods-3168882-supplementary.pdf]

Table S1 Ingredients and nutritional value of commercial concentrate

Items[1]	Proportion, %	DM, %	CP, %DM	EE, %DM	TDN, %DM	Ca, %DM	P, %DM
Soybean meal	28.00	88.00	48.86	3.86	90.91	0.03	0.32
Cottonseed meal	20.00	88.00	52.27	2.39	81.82	0.18	1.14
DDGS	12.00	90.00	28.89	9.78	86.67	0.16	0.96
Corn Gluten	13.00	88.00	21.02	2.27	82.95	0.23	1.02
Wheat bran	8.50	88.00	17.05	4.55	70.45	0.16	1.33
Palm meal	5.00	88.00	17.05	10.80	85.23	0.13	0.67
Sesame meal	4.00	91.00	49.45	3.30	76.92	2.20	1.43
Limestone	4.00	99.00	0.00	0.00	0.00	38.00	0.00
$NaHCO_3$	1.00	99.00	0.00	0.00	0.00	0.00	0.00
MgO	0.50	99.00	0.00	0.00	0.00	0.00	0.00
Molasses	2.00	60.00	5.00	0.00	90.00	1.37	0.13
1%Premix[2]	2.00	99.00	0.00	——	——	3.03	1.01
Total	100.00	88.63	34.58	4.08	77.34	2.01	0.79

[1]DM, dry matter; CP, crude protein; EE, ether extract; NDF, neutral detergent fiber; ADF, acid detergent fiber; TDN, Total digestible nutrients.

[2]The 1%premix contained the following per kg of diet: Cu 3150 mg、Fe 3000 mg、Zn 13750 mg、Mn 14250 mg、I 190 mg、Co 125 mg、Se 100 mg、VA 1792800

IU、VD3 478000 IU、VE 7968 mg.

Table S2 Effect of PBP replacing corn in different proportions on growth performance of Angus bulls

Ingredients		PBP to replace corn in different proportions[1]				SEM	*P*-value		
		0% PBP	12.84% PBP	25.65% PBP	38.44% PBP		Treatment	Linear	Quadratic
BW[2], kg	0d	548.07	548.17	548.90	548.73	5.620	0.998	0.878	0.973
	31d	624.97	620.80	617.86	614.37	0.456	0.600	0.177	0.952
	61d	688.87	682.97	684.04	673.96	3.213	0.450	0.144	0.766
	91d	727.33[a]	724.00[a]	717.58[ab]	708.17[b]	2.132	0.003	＜0.001	0.373
0～30d	DMI, kg	12.38	12.38	12.38	12.38	—	—	—	—
	ADG, kg	1.97	1.96	1.77	1.68	0.057	0.327	0.067	0.928
	FCR	6.52	6.99	7.40	8.45	0.322	0.188	0.035	0.651
31~60d	DMI, kg	14.15	14.15	14.15	14.15	—	—	—	—
	ADG, kg	1.99	1.94	2.07	1.93	0.048	0.740	0.861	0.666
	FCR	6.99	7.37	7.55	7.73	0.206	0.624	0.778	0.505

61~90d	DMI, kg	15.06	15.06	15.06	15.06	—	—	—	—
	ADG, kg	1.53	1.48	1.31	1.32	0.050	0.272	0.068	0.778
	FCR	10.36	11.67	12.30	14.44	0.932	0.492	0.314	0.364
0~90d	DMI, kg	13.73	13.73	13.73	13.73	—	—	—	—
	ADG, kg	1.88	1.80	1.74	1.70	0.028	0.108	0.015	0.768
	FCR	7.42	7.71	7.93	8.24	0.122	0.115	0.016	0.991

[1] 0% PBP, control group; 12.84% PBP, 25% replacement group; 25.65% PBP, 50% replacement group; 38.44% PBP, 75% replacement group.

[2] BW, body weight; DMI, dry matter intake; ADG, average daily gain; FCR, feed conversion ratio

Table S3 Effect of PBP replacing corn in different proportions on ruminal microbiota at the phylum level

Items	0% PBP	12.84% PBP	25.65% PBP	38.44% PBP	SEM	*P*-value
Actinobacteriota	0.0241	0.0156	0.0196	0.0063	0.0044	0.605
Bacteroidota	0.4314	0.4585	0.4823	0.4809	0.0162	0.697
Cyanobacteria	0.0008	0.0020	0.0007	0.0006	0.0003	0.286
Desulfobacterota	0.0022	0.0036	0.0019	0.0029	0.0004	0.542
Elusimicrobiota	0.0001	0.0017	0.0001	0.0001	0.0003	0.238
Fibrobacterota	0.0024	0.0123	0.0108	0.0191	0.0026	0.214
Firmicutes	0.4090	0.4288	0.3871	0.4025	0.0123	0.668
Patescibacteria	0.0097	0.0055	0.0071	0.0082	0.0012	0.682
Proteobacteria	0.1067	0.0659	0.0868	0.0725	0.0105	0.566
Spirochaetota	0.0009	0.0021	0.0013	0.0023	0.0003	0.344

Synergistota	0.0006	0.0012	0.0008	0.0010	0.0001	0.485
Verrucomicrobiota	0.0049	0.0013	0.0004	0.0023	0.0007	0.123
unclassified_Bacteria	0.0035	0.0012	0.0008	0.0011	0.0006	0.412

[1] 0% PBP, control group; 12.84% PBP, 25% replacement group; 25.65% PBP, 50% replacement group; 38.44% PBP, 75% replacement group.

Table S4 Effect of PBP replacing corn in different proportions on ruminal microbiota at the genus level

Items	PBP to replace corn in different proportions[1]				SEM	*P*-value		
	0% PBP	12.84% PBP	25.65% PBP	38.44% PBP		Treatment	Linear	Quadratic
Bifidobacterium	0.0139	0.0133	0.0177	0.0047	0.0036	0.664	0.507	0.41
NK4A214_group	0.0101	0.0149	0.0112	0.0152	0.0012	0.392	0.338	0.864
Prevotella	0.2790	0.2153	0.2479	0.2741	0.0188	0.385	0.922	0.255
Rikenellaceae_RC9_gut_group	0.0249	0.0339	0.0320	0.0367	0.0032	0.663	0.285	0.747
Saccharofermentans	0.0221	0.0152	0.0108	0.0190	0.0023	0.348	0.530	0.112
Shuttleworthia	0.0231	0.0121	0.0164	0.0014	0.0038	0.299	0.100	0.800
Succinivibrio	0.0114	0.0160	0.0093	0.0049	0.0024	0.191	0.251	0.352
Succinivibrionaceae_UCG_001	0.0196	0.0120	0.0099	0.0007	0.0043	0.559	0.164	0.923
Succinivibrionaceae_UCG_002	0.0152	0.0112	0.0308	0.0290	0.0039	0.167	0.103	0.891
UCG_002	0.0103	0.0262	0.0190	0.0200	0.0033	0.429	0.490	0.272
[Eubacterium]_ruminantium_group	0.0106	0.0141	0.0082	0.0076	0.0015	0.770	0.295	0.507
unclassified_Clostridia	0.0093	0.0140	0.0111	0.0154	0.0012	0.298	0.175	0.936
unclassified_Clostridia_UCG_014	0.0102	0.0102	0.0178	0.0141	0.0021	0.489	0.330	0.661
unclassified_Succinivibrionaceae	0.0078	0.0079	0.0228	0.0221	0.0039	0.316	0.118	0.961

[1] 0% PBP, control group; 12.84% PBP, 25% replacement group; 25.65% PBP, 50% replacement group; 38.44% PBP, 75% replacement group.

Table S5 Effect of PBP replacing corn in different proportions on economic benefits

Ingredients	PBP to replace corn in different proportions				SEM	*P*-value		
	0% PBP	12.84% PBP	25.65% PBP	38.44% PBP		Treatment	Linear	Quadratic
Daily feed cost[1], CNY[4]/head	38.6	34.0	29.4	24.8	—	—	—	—
Feed cost per weight gain[2], CNY/kg	20.84[a]	19.11[b]	16.99[c]	14.88[d]	0.393	＜0.001	＜0.001	0.728
Farm Profit[3], CNY/head	2640.09	2822.05	3060.99	3353.61	96.382	0.051	0.006	0.768

[1]Feed cost was based on the actual market prices of various ingredients during the trial period；

[2]Feed cost per weight gain = Daily feed cost / ADG;

[3]Farm Profit = Total weight gain during the test period * Average actual selling price of cattle (35.0 CNY/kg) - Total feed cost.

[4]CNY, Chinese Yuan.

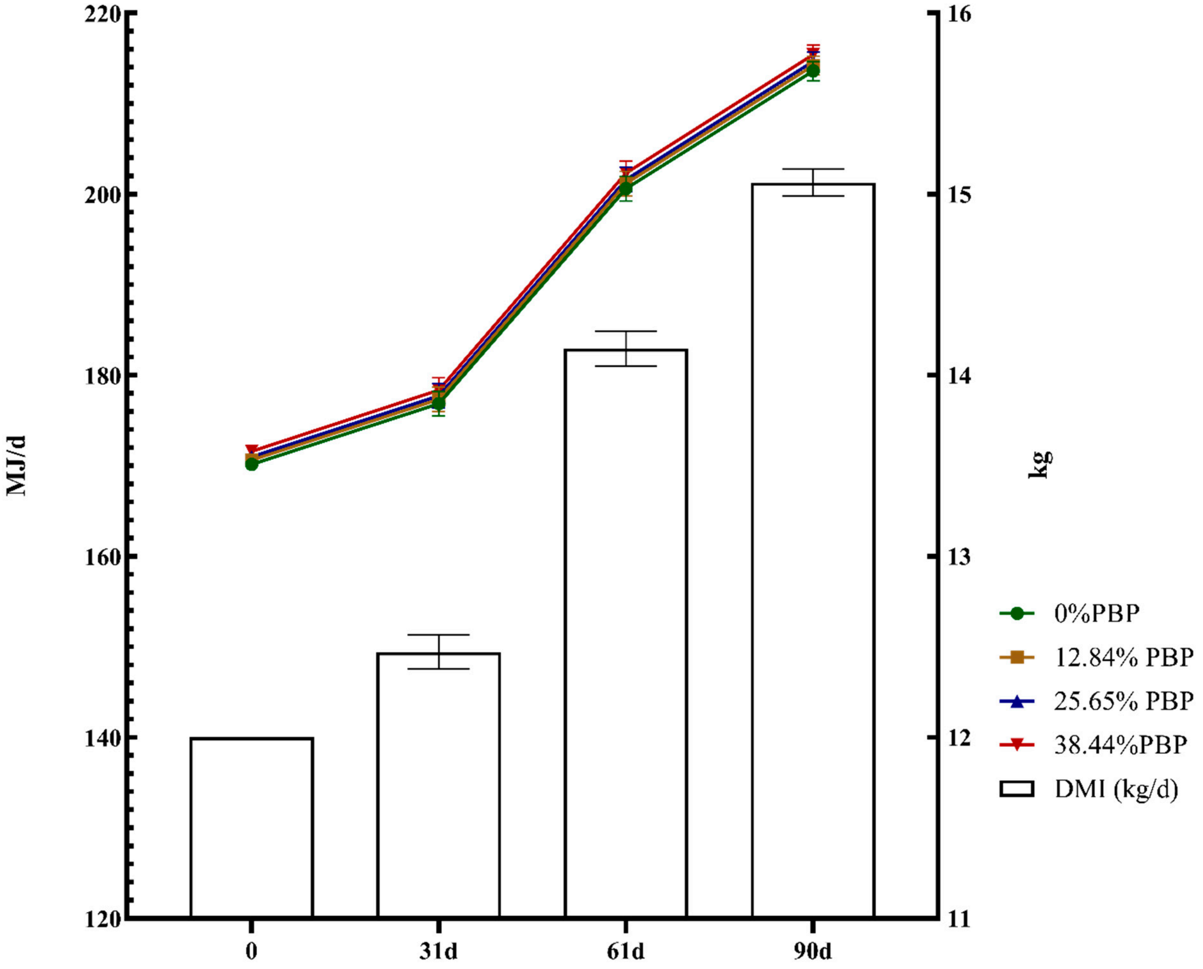

Figure S1. The changes in DMI (dry matter intake) and MEI (metabolizable energy intake) of Angus bulls during the experimental period

A

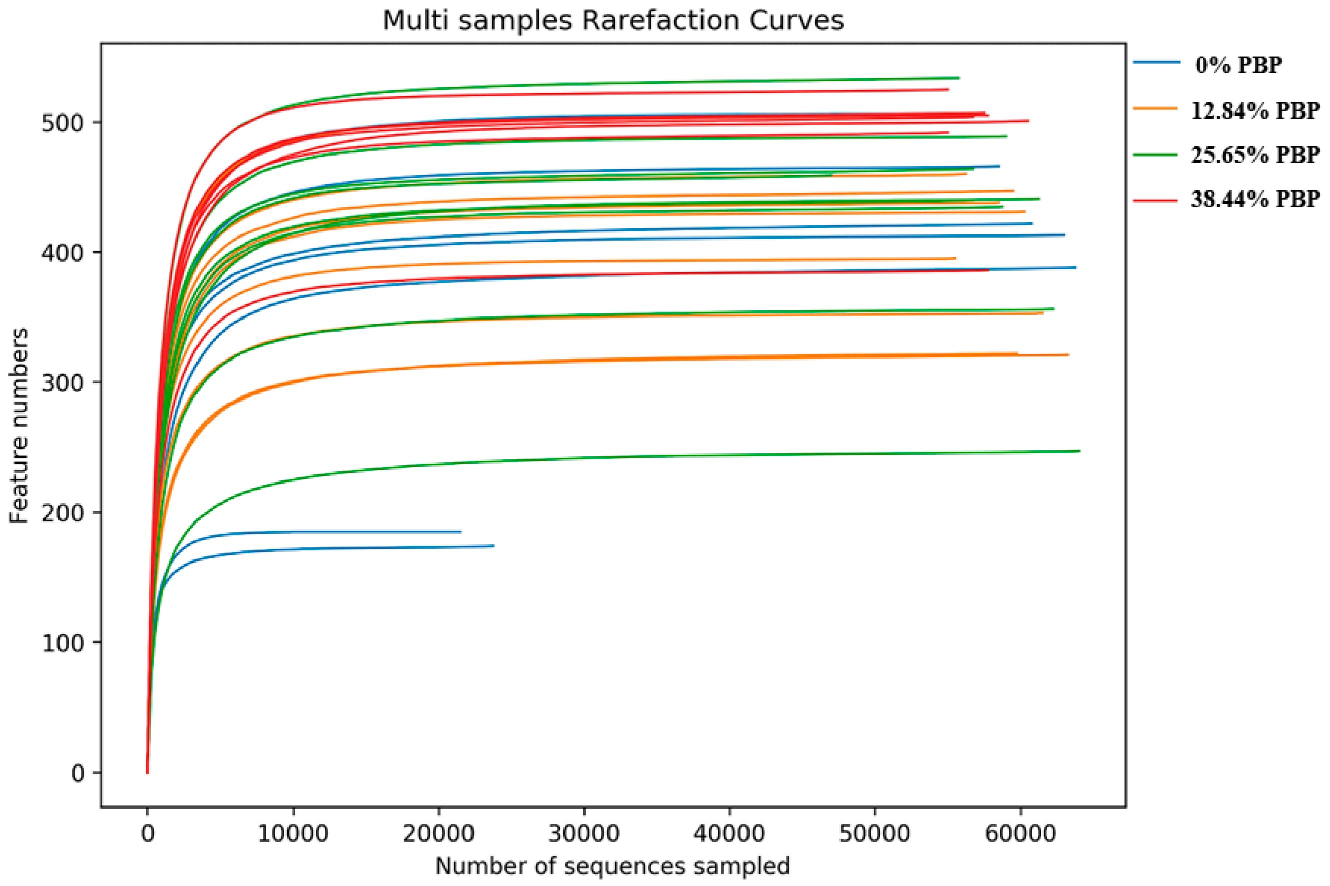

Multi samples Rarefaction Curves
0% PBP
12.84% PBP
25.65% PBP
38.44% PBP
Feature numbers
0
100
200
300
400
500
0
10000
20000
30000
40000
50000
60000
Number of sequences sampled

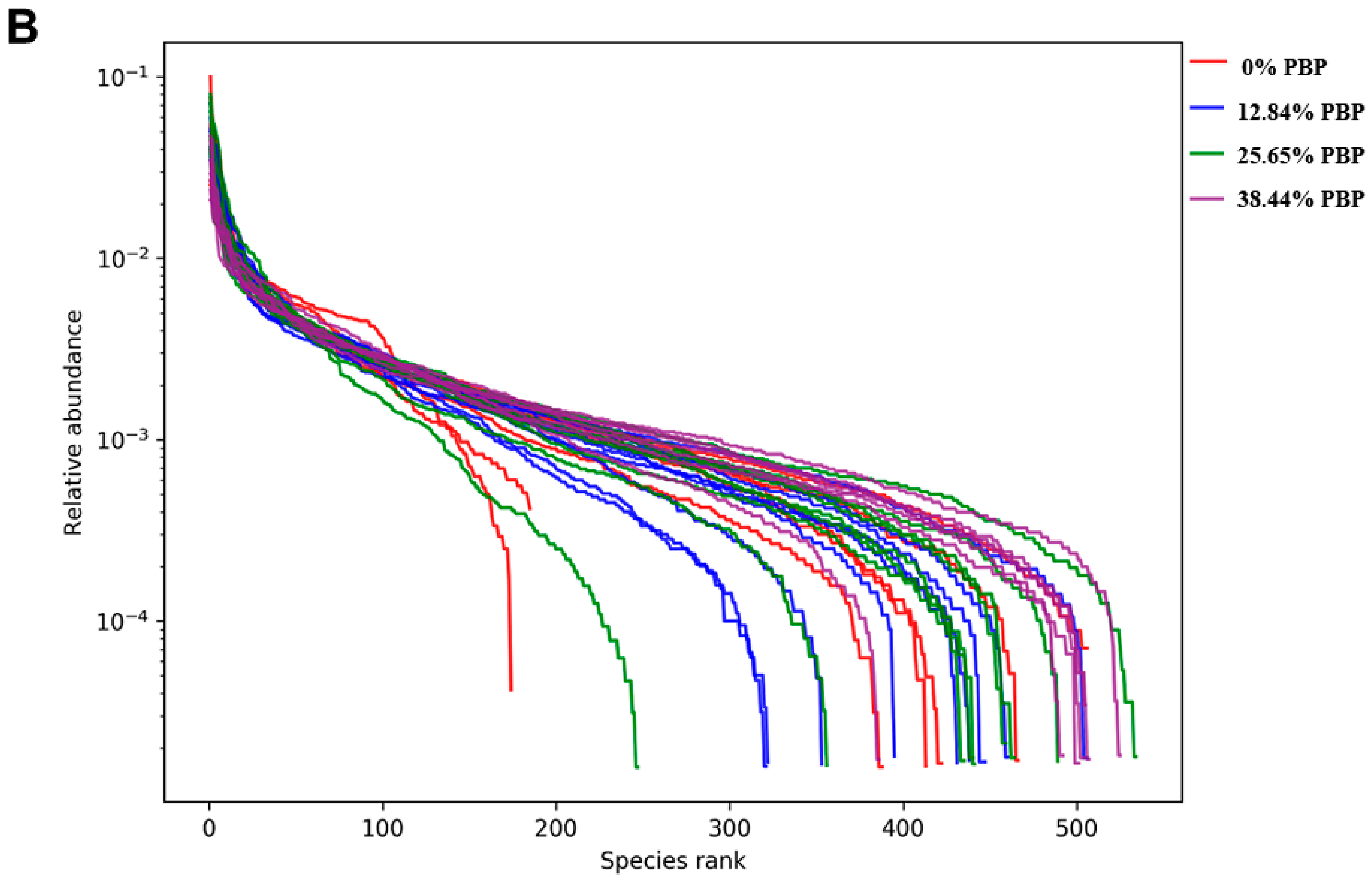

B
Relative abundance
Species rank
0% PBP
12.84% PBP
25.65% PBP
38.44% PBP

C

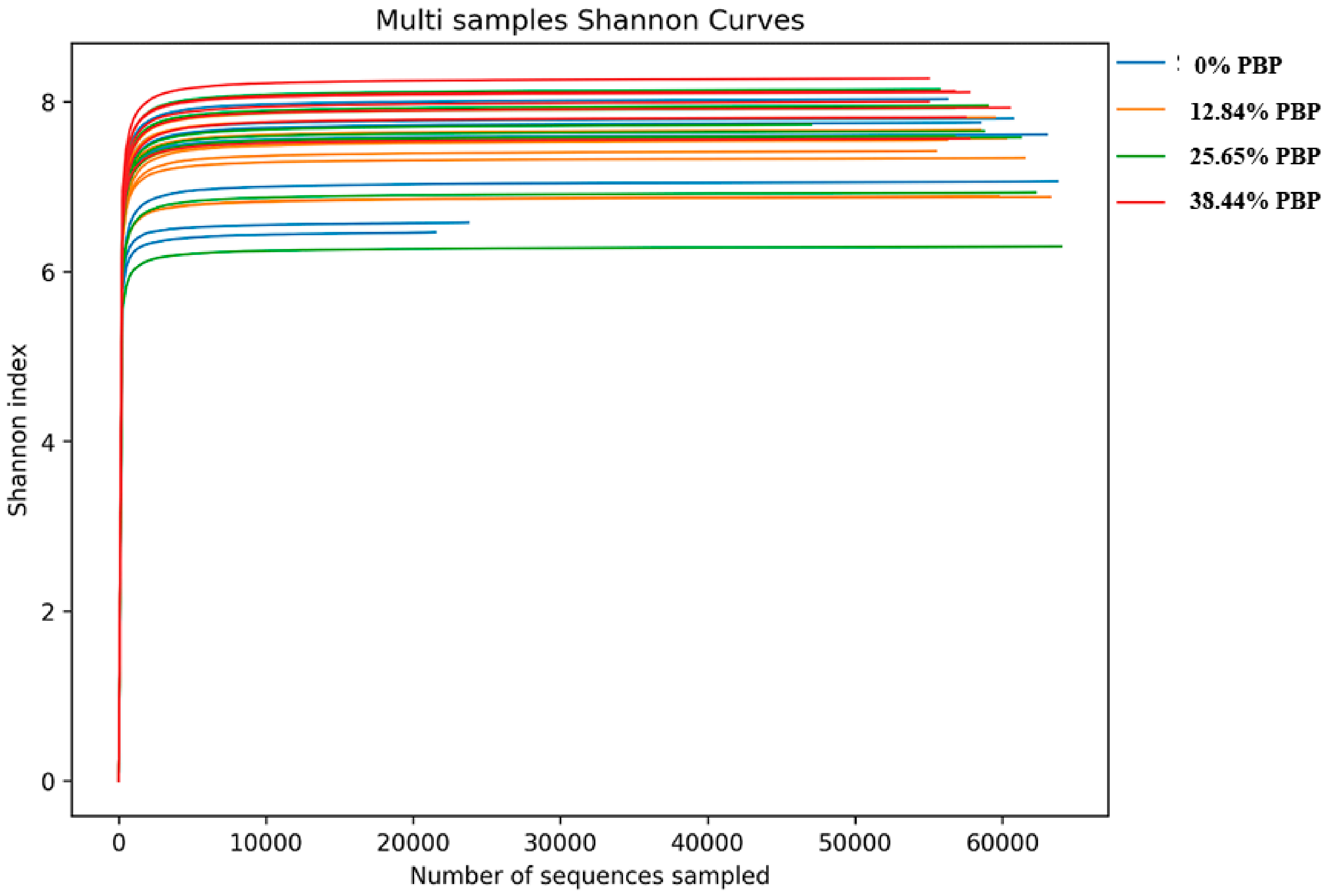

Figure S2 *16S r*RNA gene analysis of bacterial communities
